# Hypervirulent Clone of Group B *Streptococcus* Serotype III Sequence Type 283, Hong Kong, 1993–2012

**DOI:** 10.3201/eid2210.151436

**Published:** 2016-10

**Authors:** Margaret Ip, Irene Ang, Kitty Fung, Veranja Liyanapathirana, Ming Jing Luo, Raymond Lai

**Affiliations:** Chinese University of Hong Kong, Hong Kong, China; and Prince of Wales Hospital, Hong Kong

**Keywords:** Group B Streptococcus, serotype III-4/ST283, newborns, hypervirulent, clone, meningitis, fish disease, zoonoses, Hong Kong, bacteria

## Abstract

We describe a hypervirulent clone of group B *Streptococcus* serotype III, subtype 4, sequence type 283, that caused invasive disease with a predilection for meningitis in Hong Kong during 1993–2012. The organism is associated with high mortality and increased summer prevalence and is linked to diseased fish from freshwater fish farms.

Group B S*treptococcus* (GBS) has been implicated in neonatal sepsis and infections in pregnant women. Worldwide, the predominant clone belongs to serotype III, subtype 2 (III-2), sequence type (ST) 17 (*1*), which is less genetically diverse and heterogeneous than strains from the other serotypes (Ia, Ib, II–IX). In July 2015, the Singapore Ministry of Health reported an outbreak of GBS invasive disease in adults and suspected a link to raw fish consumption (*2*). The Ministry subsequently reported that this GBS belonged to ST283 (*3*), and its whole genome has been recently sequenced (*4*). GBS strains of ST283 were linked to a series of adult meningitis cases in Singapore and Hong Kong dating from 1998 (*5*). In addition, GBS III-4/ST283 is a hypervirulent lineage that has been associated with invasive GBS disease in nonpregnant adults (*6*). We investigated the epidemiology of GBS infections caused by GBS III-4/ST283 during 1993–2012 in Hong Kong and examined possible associations with seasons, temperature, and humidity. We also compared the invasive potential of different GBS serotypes among neonates, pregnant women, and nonpregnant adults. 

## The Study

We investigated GBS serotypes found among 1,645 isolates from patients admitted to a university hospital in Hong Kong during 1993–2012. Of the isolates, 437 were invasive GBS (i.e., isolated from sterile blood, cerebrospinal fluid, and body fluid specimens), and 1,208 were noninvasive (Technical Appendix Table). We stratified these patients into 3 groups: 249 neonates, 704 pregnant women, and 692 nonpregnant adults (women and adults were >16 years of age). We used PCR, pulsed-field gel electrophoresis, and multilocus sequence typing for serotyping and subtyping of the isolates, as described (*1**,**6*). We reviewed demographic and medical records for the 1,645 patients and performed statistical analysis by using Fisher exact and χ^2^ tests. SPSS version 19 (IBM, Armonk, New York, USA) was used for Spearman correlation analysis. We obtained ethical approval from the Joint Chinese University of Hong Kong–New Territories East Cluster Clinical Research Ethics Committee (CRE-2012.054).

Using an empirical odds ratio (OR) (*7*), we compared the invasive potential of serotype III-4 isolates with that of all other serotypes (Table 1). Serotypes III-2 (n = 76; OR 1.5, 95% CI 1.1–2.1; p<0.01) and III-4 (n = 50; OR 19.4, 95% CI 9.1–41.2; p<0.001) had significantly higher (OR >1.0) invasive potential than other serotypes. Serotypes III-2 was the predominant cause of invasive disease in neonates (OR 5.3, 95% CI 2.7–10.3; p<0.001). Serotype III-4 was highly invasive in nonpregnant adults (OR 18.4, 95% CI 7.7–43.9; p<0.001) and in neonates (OR 6.3, 95% CI 0.7–54.3; p<0.01).

**Table 1:** Invasive potential of individual group B *Streptococcus* serotypes among neonates, nonpregnant adults and pregnant women, Hong Kong, 1993–2012*

Serotype	Odds ratio (95% CI), p value			
	All patients, n = 1,645	Neonates, n = 249	Nonpregnant adults, n = 692	Pregnant women, n = 704
Ia	**0.6 (0.4–0.8), <0.001**	0.7 (0.4–1.4), 0.37	**0.4 (0.2–0.6), <0.001**	1.0 (0.6–1.6), 0.94
Ib	1.0 (0.7–1.3), 0.92	1.1 (0.5–2.5, 0.74	1.2 (0.8–1.8), 0.30	0.7 (0.4–1.3), 0.30
II	0.9 (0.6–1.3), 0.49	**0.2 (0.1–0.5), <0.01**	0.9 (0.5–1.6), 0.76	**2.0 (1.0–3.7), <0.04**
III-1	1.1 (0.8–1.5), 0.69	1.2 (0.6–2.3), 0.65	1.3 (0.8–2.1), 0.33	0.8 (0.4–1.6), 0.58
III-2	**1.5 (1.1–2.1), <0.01**	**5.3 (2.7–10.3), <0.001**	**0.5 (0.3–1.0), <0.05**	1.4 (0.8–2.5), 0.22
III-3	1.1 (0.4–2.9), 0.83	8.6 (0.4–169.2), 0.16	0.8 (0.2–3.3), 0.79	0.4 (0.0–6.3), 0.49
III-4	**19.4 (9.1–41.2), <0.001**	**6.3 (0.7–54.3), <0.01**	**18.4 (7.7–43.9), <0.001**	6.3 (0.4–101.8), 0.19
IV	0.1 (0.0–2.2), 0.16	0.4 (0.0–9.9), 0.57	0.2 (0.0–4.2), 0.32	0.6 (0.0–10.2), 0.70
V	**0.6 (0.5–0.9), <0.02**	**0.1 (0.0–0.4), 0.001**	0.9 (0.5–1.4), 0.60	0.8 (0.4–1.6), 0.56
VI	0.8 (0.4–1.5), 0.51	0.2 (0.0–2.0), 0.19	1.0 (0.5–2.2), 0.95	0.7 (0.4–2.8), 0.57
VII	2.3 (0.7–7.6), 0.17	0.4 (0.0–9.9), 0.57	14.5 (0.7–282.2), 0.08	2.5 (0.5–13.2), 0.27
VIII	0.1 (0.0–2.2), 0.16	0.4 (0.0–9.9), 0.57	0.2 (0.0–4.2), 0.32	0.6 (0.0–10.2), 0.70
IX	0.9 (0.0–22.7), 0.96	1.2 (0.0–61.1), 0.93	2.0 (0.0–103.5), 0.72	2.1 (0.1–51.3), 0.66
NT	1.4 (0.1–15.3), 0.79	1.2 (0.0–61.1), 0.93	2.1 (0.1–33.0), 0.61	2.1 (0.1–51.3), 0.66

*Bold indicates statistical significance; p values determined by Fisher exact or χ^2^ test.

**Figure 1:**
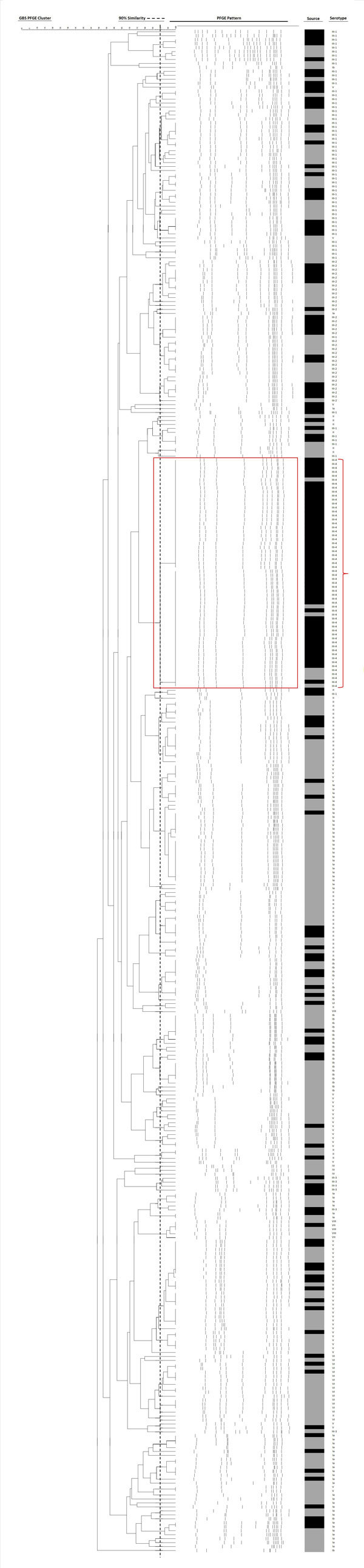
Dendrogram analysis of *Sma*I-digested pulsed-field gel electrophoresis fingerprints of 58 isolates of group B *Streptococcus* (GBS), serotype III, subtype 4 (fingerprints highlighted in red box and bracket) and of 324 randomly selected GBS isolates of different serotypes, Hong Kong, 1993–2012. The dotted vertical line on the serotype column delineates the pulsed-field gel electrophoresis clustering at 90% similarity, determined by analysis by dice coefficient with 1% tolerance and by the unweighted pair group method with arithmetic mean (BioNumerics version 7.0; Applied Maths, Sint-Martens-Latem, Belgium). On the source column, solid black indicates that the GBS strain was isolated from invasive sites; solid gray indicates that the strain was from noninvasive sites.

By using pulsed-field gel electrophoresis, all 48 serotype III-4 strains showed indistinguishable patterns and were confirmed as GBS III-4/ST283 (Figure 1). Medical records for these 48 patients (44 nonpregnant adults, 3 neonates, and 1 pregnant woman) revealed that invasive disease has been identified in nonpregnant adults since 1995 and invasive illness in neonates and pregnant women since 2009. 

Mean age of the 44 nonpregnant adults with GBS III-4/ST283 infection was 63 (range 23–96) years; half were >65 years of age. Twenty-eight (63.6%) of the 44 patients had underlying diseases (e.g., diabetes, hypertension, malignancy, chronic rheumatic heart, and gout). Serious complications developed in 35 (80%) of the 44 nonpregnant adult patients: 17 (38.6%) had sepsis, 10 (22.7%) had septic arthritis, 7 (15.9%) had meningitis, and 2 (4.5%) had infective endocarditis. Twelve (27.3%) patients died. For patients >65 years of age, presence of underlying diseases or serious complications was not statistically associated with fatal outcome (p>0.05). 

Of the 44 nonpregnant adults with GBS III-4/ST283, 41 (93.2%) had isolates from invasive sites. Sex of patients had no effect on whether the GBS disease was invasive: 20 of these patients were women and 21 were men. 

Among the total 227 nonpregnant adults with invasive GBS infection, 12 (5.5%) had meningitis (Table 2). Of these patients, 7 (58.3%) were infected with serotype III-4 alone. Compared with other GBS serotypes, GBS III-4 had significantly higher potential (p<0.001 by χ^2^ test) to cause meningitis in nonpregnant adults. 

**Table 2:** Distribution of GBS serotypes in patients with group B *Streptococcus* invasive disease, Hong Kong, 1993–2012*

Serotype	Nonpregnant adults, n = 267				Neonates, n = 113		
	No. (%)		p value		No. (%)		p value
	Meningitis†	Nonmeningitis‡			Meningitis†	Nonmeningitis‡	
Ia	1 (3.0)	32 (97.0)	0.53		4 (21.1)	15 (78.9)	0.55
Ib	1 (2.1)	46 (97.9)	0.28		2 (14.3)	12 (85.7)	0.27
II	0	17 (100.0)	0.31		0	4 (100.0)	0.22
III-1	1 (3.6)	27 (96.4)	0.66		5 (26.3)	14 (73.7)	0.56
III-2	0	13 (100.0)	0.38		16 (35.6)	29 (64.4)	0.08
III-3	0	3 (100.0)	0.68		2 (66.7)	1 (33.3)	0.11
III-4	7 (15.9)	37 (84.1)	**<0.001**		1 (20.0)	4 (80.0)	0.73
V	2 (7.1)	26 (92.9)	0.64		0	3 (100.0)	0.29
VI	0	10 (100.0)	0.44		0	1 (100.0)	0.55
VII	0	3 (100.0)	0.68		0	0	NA
VIII	0	0	NA		0	0	NA
IX	0	0	NA		0	0	NA
NT	0	1 (100.0)	0.81		0	0	NA
Total	12 (5.3)	215 (94.7)	NA		30 (26.5)	83 (74.3)	NA
*GBS, group B *Streptococcus*; NT, nontypable. Bold indicates statistical significance; p values determined by Fisher exact or χ^2^ test. †Meningitis was confirmed by cerebrospinal fluid culture of GBS. ‡Nonmeningitis was confirmed by GBS culture from sterile body site or from cerebrospinal fluid.							

Among the 3 neonates with invasive disease caused by GBS III-4/ST283, one infant had early-onset sepsis, and 2 had late-onset sepsis (1 with meningitis). Thirty neonates had meningitis, 16 (53.3%) caused by serotype III-2 and 1 (3.3%) caused by serotype III-4.

**Figure 2:**
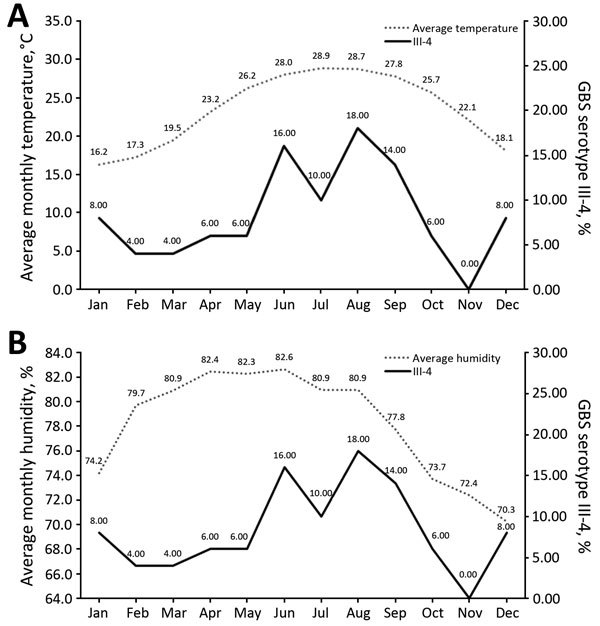
Association of temperature and humidity with distribution of isolates by month of collection from patients infected with invasive Group B *Streptococcus* (GBS) serotype III, subtype 4 (III-4), Hong Kong, 1993–2012. A) Average annual monthly temperature and distribution of invasive GBS III-4 isolates. B) Average annual monthly humidity and distribution of invasive GBS III-4 isolates. Numbers along data lines indicate monthly values.

To assess contributing factors, we examined possible climatic associations with isolation of serotypes causing the 437 cases of invasive GBS disease in Hong Kong during our study period. We reviewed Hong Kong’s average monthly temperature and humidity records for 1997–2012. Serotype III-4 was the only serotype with a prevalence significantly associated with monthly temperature (Spearman correlation r = 0.622; p = 0.031). Isolation of serotype III-4 peaked during the summer months (June–September), when mean temperature was >28°C) (Figure 2, panel A). Humidity was not significantly associated with serotype III-4 prevalence (p>0.05) (Figure 2, panel B). 

GBS infects many host species, has become an important pathogen in the aquaculture industry, and has resulted in significant economic loss (*8*). Serotype Ia/ST7 has been associated with large outbreaks of streptococcosis in cultured tilapia in China (*9*). The pathogenicity of human ST7 isolates in fish has been well established (*10*). Although no epidemiologic evidence confirms GBS as a zoonosis in human infections (*11*), comparative genome analysis revealed that cultured tilapia GBS ST7 was closely related to human ST7 strain A909. These fish and human strains share highly similar clustered regularly interspaced short palindromic repeats, prophages, virulence-associated genes, and extremely short evolutionary relationships in phylogenetic analysis (*12*).

ST283 and its single-locus variant ST491 have been detected in diseased tilapia with high death rates in Southeast Asia, where fish isolates shared the same mobile genetic elements and surface proteins as the human isolates described in this article (*8**,**13*). This similarity provides molecular evidence for the linkage of ST283 strains in humans and fish. The outbreak in Singapore showed a rise in GBS sepsis and septic arthritis, which were linked to a probable source of raw fish consumption; subsequently, health authorities advised discontinuing sales of the raw fish dish as a precaution (*2*). A previous study showed that fish consumption caused a 7.3-fold increased risk for acquiring GBS serotype Ia and Ib colonization in humans (*14*). GBS serotypes Ia, III, and V of clonal complexes (CC) 19, CC23, and CC103 from a human and a cow have been shown to infect tilapia experimentally (*15*); these cross-infections support GBS pathogenicity across different species. Epidemiologic investigations and comparative genomics on a wider scale of animal and human strains may further reveal evolutionary relationships between these GBS lineages.

Our finding of an association of the GBS III-4/ST283 lineage with summer months coincides with the timing of the outbreak in Singapore. Because GBS grows rapidly in warm temperatures, a higher infective dose may occur in warmer months than at other times. Warm temperatures might also cause increased pathogenicity of this organism, a postulation supported by the association of higher temperatures with tilapia deaths caused by GBS III-4 in freshwater fish farms (*13*). The pathogenicity of this strain in fish has been shown in vivo and further highlights the pathogenic potential of this lineage.

Reports dating from the 1990s indicate that GBS ST283 isolated from humans came exclusively from invasive sites in nonpregnant adults. Our data suggest that these isolates might have undergone adaptive evolution in recent years to colonize and invade neonates with early- and late-onset GBS sepsis. Studies addressing the changing epidemiology of this hypervirulent lineage and its relationship to humans and fish are warranted to reduce potential transmission between the 2 hosts.

Technical Appendix Serotype distribution of group B Streptococcus isolates in study of invasive disease in Hong Kong, 1993–2012.
