# Supplementary material for: Hypervirulent Clone of Group B Streptococcus Serotype III Sequence Type 283, Hong Kong, 1993–2012
Source: Emerg Infect Dis. 2016 Oct;22(10):1800–3. doi: 10.3201/eid2210.151436 (PMC5038432; doi:10.3201/eid2210.151436)
Supplement: Technical Appendix — Serotype distribution of group B Streptococcus isolates in study of invasive disease in Hong Kong, 1993–2012. [file 15-1436-Techapp-s1.pdf]

Article DOI: http://dx.doi.org/10.3201/eid2210.151436

Hypervirulent Clone of Group B *Streptococcus* Serotype III Sequence Type 283, Hong Kong, 1993–2012

Technical Appendix

Technical Appendix Table. Serotype distribution of 1,645 Group B *Streptococcus* isolates (437 invasive [from sterile body sites] and 1,208 from noninvasive sites) for neonates, nonpregnant adults, and pregnant women, Hong Kong, 1993–2012*

Infection type and population	Serotype, no. (%)														
	Ia	Ib	II	III-1	III-2	III-3	III-4	IV	V	VI	VII	VIII	IX	NT	Total
Invasive															
Total	75 (17)	75 (17)	35 (8)	59 (14)	76 (17)	6 (1)	50 (11)	0	42 (10)	13 (3)	5 (1)	0	0	1 (0)	437
Neonates	19 (17)	14 (12)	4 (4)	19 (17)	45 (40)	3 (3)	5 (4)	0	3 (3)	1 (1)		0	0	0	113
Nonpregnant adults	33 (15)	47 (21)	17 (7)	28 (12)	13 (6)	3 (1)	44 (19)	0	28 (12)	10 (4)	3 (1)	0	0	1 (0)	227
Pregnant women	23 (24)	14 (14)	14 (14)	12 (12)	18 (19)		1 (1)	0	11 (11)	2 (2)	2 (2)	0	0	0	97
Noninvasive															
Total	321 (27)	210 (17)	110 (9)	154 (13)	147 (12)	15 (1)	8 (1)	10 (1)	170 (14)	44 (4)	6 (0)	10 (1)	1 (0)	2 (0)	1,208
Neonates	29 (21)	15 (11)	24 (18)	20 (15)	15 (11)	0	1 (1)	1 (1)	24 (18)	5 (4)	1 (1)	1 (1)	0	0	136
Nonpregnant adults	146 (31)	81 (17)	38 (8)	46 (10)	48 (10)	7 (2)	6 (1)	4 (1)	64 (14)	20 (4)		4 (1)	0	1 (0)	465
Pregnant women	146 (24)	114 (19)	48 (8)	88 (14)	84 (14)	8 (1)	1 (0)	5 (1)	82 (14)	19 (3)	5 (1)	5 (1)	1 (0)	1 (0)	607
Total	396	285	145	213	223	21	58	10	212	57	11	10	1	3	1,645

*NT, nontypable. Number in parentheses indicates percentage of specific serotypes for each patient group.
